# Ergothioneine: An Antioxidative, Neuroprotective and Anti-Inflammatory Compound from Mushroom Residuals

**DOI:** 10.3390/molecules30234621

**Published:** 2025-12-01

**Authors:** Joanna Harasym, Alona Tiupova, Ewa Pejcz

**Affiliations:** 1 Department of Biotechnology and Food Analysis, Wroclaw University of Economics and Business, Komandorska 118/120, 53-345 Wroclaw, Poland; alona.tiupova@ue.wroc.pl (A.T.); ewa.pejcz@ue.wroc.pl (E.P.); 2 Adaptive Food Systems Accelerator-Science Centre, Wroclaw University of Economics and Business, Komadorska 118/120, 53-345 Wroclaw, Poland

**Keywords:** ergothioneine, neuroprotection, anti-inflammatory, mushroom by-products, sustainable extraction, circular bioeconomy, oxidative stress

## Abstract

In vitro and in vivo evidence demonstrates that EGT exerts neuroprotective effects through multiple mechanisms: scavenging reactive oxygen species, suppressing neuroinflammatory cytokines (TNF-α, IL-1β, IL-6), activating Nrf2 antioxidant pathways, and preserving mitochondrial integrity. Low blood EGT levels correlate with cognitive decline and dementia, supporting its role as a conditionally essential micronutrient for healthy aging. Mushroom by-products retain EGT concentrations comparable to commercial fruiting bodies, making them viable sources for dietary supplements and functional foods. Mushroom processing generates substantial residual biomass—including stems, culls, and spent substrate—that represents an underexploited dietary source of ergothioneine (EGT), a naturally occurring antioxidant with exceptional neuroprotective and anti-inflammatory properties. Since humans cannot synthesize EGT endogenously, dietary intake is essential for maintaining neuroprotection against neurodegenerative diseases. This review examines sustainable extraction strategies—including hot-water, ultrasound-assisted, and high-hydrostatic-pressure methods—enabling integration into circular biorefinery systems. Applications in nutraceuticals and pharmaceuticals targeting oxidative stress-related neurodegeneration are highlighted. Despite challenges in standardization and regulatory approval, valorizing mushroom residuals offers a sustainable pathway to increase dietary availability of this neuroprotective antioxidant, supporting both environmental sustainability and therapeutic innovation for neurodegenerative disease prevention.

## 1. Introduction

Ergothioneine (EGT) is a unique, naturally occurring sulfur-containing derivative of histidine, chemically designated as (2S)-3-(2-thioxo-2,3-dihydro-1H-imidazol-4-yl)-2-(trimethylammonio) propanoate [1,2]. Its remarkable stability arises from its predominant “thione” tautomeric form at physiological pH, which confers exceptional resistance to autoxidation and degradation, even under conditions of elevated temperature or variable pH [2]. This chemical resilience enhances its suitability for integration into food systems, cosmetic formulations, and pharmaceutical products, ensuring long-term bioactivity and stability. As a hydrophilic zwitterion, EGT exhibits specific solubility and extraction behavior, influencing the selection of optimal recovery methods [3].

A defining aspect of EGT’s biological relevance lies in its active transport via the organic cation transporter OCTN1 (SLC22A4) [1,2,4,5,6]. This highly selective mechanism facilitates its accumulation in tissues particularly susceptible to oxidative stress, such as erythrocytes, liver, kidneys, and the eye lens, underscoring its critical physiological role in cellular protection [1,2].

Among dietary sources, mushrooms are unequivocally recognised as the richest, accounting for approximately 95% of total intake in typical diets [7,8,9,10,11,12,13]. Other foods, such as meat, seafood, grains, and vegetables, contain only trace amounts [7]. EGT is synthesised exclusively by certain microorganisms, primarily fungi and actinobacteria [1,2,5,6,7]. Plants may acquire it from symbiotic interactions with these microorganisms in the rhizosphere [1,2]. The existence of a dedicated transporter for EGT, coupled with its absence of endogenous synthesis and the association of low plasma levels with disease risk, indicates that EGT may represent a conditionally essential micronutrient [1,5,7,8,11]. Therefore, the identification and sustainable exploitation of mushroom-based sources of EGT is essential to improving dietary availability and promoting long-term health.

The global mushroom cultivation and processing sector has expanded rapidly in response to the rising demand for edible and medicinal mushrooms; however, this growth generates considerable amounts of waste. Processing operations produce residuals, such as stems, stipes, deformed fruiting bodies, and spent mushroom substrate (SMS), which consists mainly of lignocellulosic materials remaining after harvest. These residuals may account for up to 20% of total production, and their disposal without recovery contributes to greenhouse gas emissions and nutrient loss, resulting in environmental and economic inefficiencies [13,14,15]. Given their high organic and bioactive content, these residues represent a valuable but underexploited biomass stream.

Among the bioactive molecules present in mushroom residuals, EGT is particularly significant due to its potent antioxidant and cytoprotective activities. It accumulates not only in fruiting bodies but also in stalks and SMS, making these by-products a promising alternative source for its extraction [8]. Given its physiological importance and stability, EGT recovered from mushroom waste streams can be utilized in nutraceutical, cosmetic, and pharmaceutical applications [13]. The valorization of mushroom processing residuals for EGT recovery thus represents an integrative approach linking food sustainability, environmental protection, and human health promotion [15].

While the stalks and stems of species such as *Agaricus bisporus*, *Pleurotus ostreatus*, and *Lentinula edodes* are often discarded, they contain residual nutrients and bioactive compounds, including EGT, which could be recovered for functional applications. Similarly, SMS retains fungal metabolites and mycelial biomass that can be valorized through biotechnological processing [14]. Sustainable management of these by-products aligns with global goals for responsible production and consumption (UN SDG 12) and supports the zero-waste approach to food bioprocessing, forming part of broader strategies for developing a circular bioeconomy.

Waste valorisation is a central element of the circular economy, emphasising resource efficiency by minimising waste and promoting the recovery of valuable compounds. In mushroom processing, valorisation strategies include converting residual biomass into ingredients for food, pharmaceutical, and agricultural applications, and reusing lignocellulosic materials as substrates for secondary cultivation or bioenergy generation. For example, aqueous extracts of oyster mushroom by-products enhance the growth of probiotic bacteria, whereas antimicrobial and antioxidant components from shiitake residues can serve as natural preservatives [16]. Agricultural co-residues, such as sugarcane bagasse and banana leaves, have also been utilised as substrates for mushroom hybrids, enhancing both yield and nutritional profile [17]. Such practices not only reduce environmental impact but also contribute to economic diversification and sustainability within the agro-industrial sector.

This review aims to consolidate current evidence on the occurrence and recovery of ergothioneine from mushroom processing residuals, with a particular focus on sustainable extraction approaches and their potential application in industrial and circular bioeconomy contexts.

## 2. Ergothioneine Bioactivity

**Figure 1:**
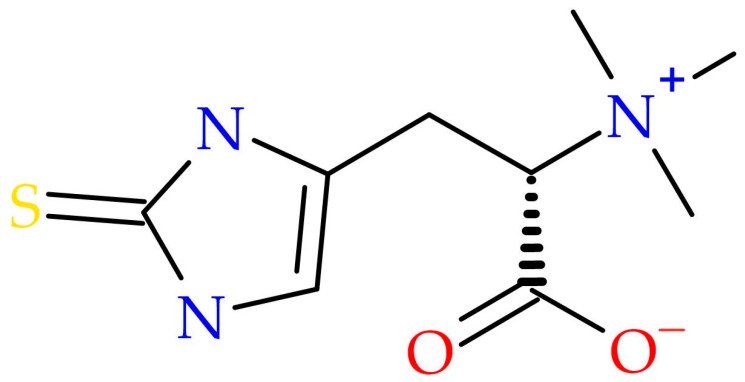
Ergothioneine molecular structure.

EGT functions as an adaptogenic antioxidant capable of scavenging reactive oxygen species (ROS) and reducing oxidative stress, thereby preventing biomolecular damage [2,3,7,8,9]. Notably, it accumulates in mitochondria—organelles highly vulnerable to oxidative injury—suggesting a central role in maintaining mitochondrial integrity [2,3]. The ergothioneine molecular structure is given in Figure 1.

Beyond its antioxidative action, EGT exhibits anti-inflammatory, neuroprotective, and cardioprotective activities [18,19,20]. Low blood levels of EGT have been correlated with cognitive decline, dementia, and increased cardiovascular risk, supporting its significance in maintaining metabolic and neurological health [8,21,22]. Humans and other animals lack the enzymatic pathways necessary for endogenous EGT biosynthesis [1,2,21,22,23,24]. Thus, dietary intake is the sole determinant of its physiological availability [25].

### Antioxidant Mechanism of Ergothioneine

Ergothioneine (EGT) is a naturally occurring thiol-histidine derivative that exhibits a broad spectrum of antioxidant and cytoprotective activities. Its cellular uptake is mediated by the highly specific organic cation transporter OCTN1 (encoded by the *SLC22A4* gene), which facilitates accumulation of EGT within mammalian cells even against a concentration gradient. Once internalised, EGT exists predominantly in its thione tautomeric form, which is chemically stable and resistant to autooxidation under physiological conditions [26]. EGT performs its antioxidant function via several complementary mechanisms. First, it directly scavenges reactive oxygen species (ROS), including hydroxyl radicals (•OH), singlet oxygen, hydrogen peroxide (H_2_O_2_), hypochlorous acid (HOCl), and peroxynitrite (ONOO^−^), thereby reducing oxidative damage to lipids, proteins, and nucleic acids [27,28,29].

**Figure 2:**
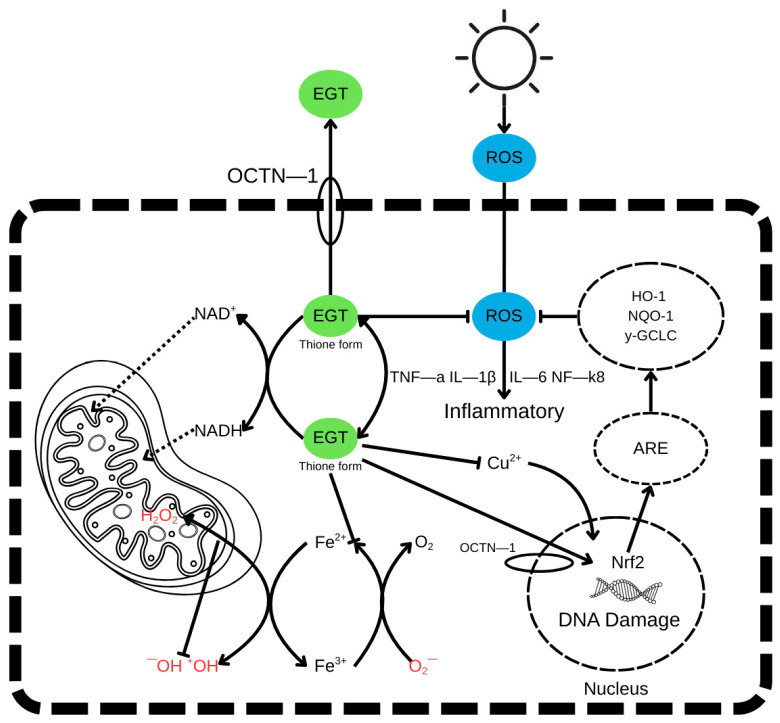
The antioxidant mechanism of ergothioneine. Green—EGT—ergothioneine, Blue—ROS—radical oxygen species.

The antioxidant mechanism of ergothioneine, highlighting the thione–thiol tautomerism and key reactive sites responsible for its redox stability and radical-scavenging activity, is shown in Figure 2.

Beyond direct ROS neutralisation, EGT modulates key cellular pathways involved in oxidative stress response and inflammation. It suppresses the expression of proinflammatory cytokines, including tumour necrosis factor-alpha (TNF-α), interleukin-1 beta (IL-1β), and interleukin-6 (IL-6), partly through inhibition of the NF-κB signalling pathway. EGT also activates the Nrf2 (nuclear factor erythroid 2-related factor 2) pathway, a master regulator of antioxidant defence. Upon activation, Nrf2 translocates to the nucleus, binds to antioxidant response elements (AREs) in DNA, and induces the expression of cytoprotective genes such as heme oxygenase-1 (HO-1), NAD(P)H quinone dehydrogenase 1 (NQO1), and γ-glutamylcysteine ligase catalytic subunit (γ-GCLC) [2,3,26].

This multi-targeted action of EGT contributes to the protection of mitochondrial function, the prevention of DNA damage, the inhibition of apoptosis, and the maintenance of redox homeostasis in stressed or ageing cells. The coordinated interaction of radical scavenging, metal chelation, anti-inflammatory signalling, and transcriptional activation positions EGT as a highly effective endogenous antioxidant with therapeutic potential in oxidative stress-related pathologies [30].

## 3. Mushroom Processing Residuals as an EGT Source

### 3.1. The Types of Mushroom Processing Residuals

The mushroom industry, while a significant contributor to global food supply, generates substantial quantities of organic waste during cultivation and processing. These residuals primarily include spent mushroom substrate (SMS), the leftover lignocellulosic material after harvesting, as well as stems and culls (low-grade or unharvested materials) [21,31,32]. The scale of this waste is considerable, with over 100 million tons of SMS produced annually worldwide, posing a major environmental challenge if not properly managed [21]. Mushroom stems, often discarded due to their tougher texture and perceived lower culinary value compared to caps, constitute a large proportion of this waste stream [31].

Quantitative analyses confirm that these processing residuals, including stalks, stems, and spent substrates, retain substantial levels of ergothioneine comparable to, or even exceeding, those found in market-ready mushroom fruiting bodies. Such data highlight that mushroom waste materials are not merely by-products but valuable reservoirs of bioactive compounds that can be recovered for functional and industrial use [8,21,31].

### 3.2. Ergothioneine Content in Mushroom Species and Residuals

Mushrooms are exceptionally rich in ergothioneine (EGT), with concentrations typically ranging from 0.1 to 1 mg/g in dried material [25]. However, certain species demonstrate considerably higher levels. Ergothioneine content in various mushroom species and parts is summarized in Table 1.

Crucially, mushroom processing residuals also contain a significant amount of EGT. Stems often exhibit equal or higher concentrations of EGT and other antioxidants compared to caps [21,31,33]. In some studies, oyster mushroom stem extracts have shown greater antioxidant activity than cap extracts [33]. High EGT levels have been reported in culls of shiitake and grey oyster mushrooms, matching those in marketable fruiting bodies [32,33]. Even *Agaricus bisporus* (white button) stem waste retains EGT contents comparable to whole mushrooms [33]. Moreover, SMS used for cultivating high-EGT species can itself become enriched with EGT; for example, *Pleurotus pulmonarius* grown on SMS reached 2.17 mg/g dry matter [18]. Mushrooms cultivated using food waste as a culture medium also show increased EGT accumulation [21], indicating that substrate composition directly affects both mushroom and residual EGT levels. Table 1 summarizes EGT concentrations across different mushroom species and parts, providing a comparative overview between fruiting bodies and processing residuals.

### 3.3. Factors Influencing EGT Content in Mushrooms and Residuals

EGT distribution varies among different parts of the mushroom and at various growth stages. While some studies report similar concentrations in caps and stems [21,25,33], others indicate a tendency for accumulation in upper tissues as mushrooms mature [25]. Mycelial cultures can even surpass fruiting bodies in EGT content when optimized conditions are applied [25,36].

The EGT content in mushrooms and their residuals is influenced by multiple factors, including cultivation practices and post-harvest processing. Variations arise from the choice of cultivation substrate (e.g., food waste versus sawdust), soil health, and tillage methods [21,25]. Supplementing growth media with amino acids such as histidine, methionine, and cysteine markedly enhances EGT synthesis [21,25,36]. For example, supplementation with 0.5 mM methionine, cysteine, and histidine, combined with yeast extract and peptone, has proven highly effective for Agaricus species [25,36]. Similar enhancements were achieved in *Panus conchatus* cultures [18]. Light conditions also influence biosynthesis, with blue LED irradiation during development shown to increase EGT content [21].

Storage and processing further impact EGT retention. EGT in mushroom caps decreases during storage, particularly at elevated temperatures, emphasising the importance of low-temperature preservation [23,32,33]. Among drying techniques, freeze-drying and hot-air drying at 40 °C show favourable retention, while natural ventilation drying can even enhance amino acid metabolism and EGT accumulation [32,33]. Microwave-vacuum drying is also effective due to the reduced boiling point under vacuum [32]. Conversely, boiling causes substantial EGT loss through leaching, while steaming better preserves it [33,35]. The choice of extraction solvent is also crucial; for instance, 70% ethanol efficiently recovers EGT and phenolics from *Flammulina velutipes* [33], whereas hot water extraction may yield higher EGT contents in some species [25].

### 3.4. Variability of Ergothioneine Content: Effects of Drying and Species Differences

Ergothioneine (EGT) in mushrooms is relatively robust to mild dehydration; however, its retention is strongly dependent on the drying regime and the species. In *Pleurotus citrinopileatus*, natural-ventilation drying increased EGT to 3.72 ± 0.06 mg/g (d.w.) compared with fresh (2.63 ± 0.06 mg/g) and freeze-dried samples (2.53 ± 0.04 mg/g), whereas hot-air drying reduced EGT to 2.37 ± 0.07 mg/g (d.w.)—consistent with ongoing biosynthesis during slow drying and partial thermal loss under convective heating [39]. Table 2 summarises published data on the effects of various drying methods and processing conditions on ergothioneine (EGT) retention in different mushroom species, highlighting interspecies variability and the influence of dehydration parameters on EGT stability.

Across species, freeze-drying generally preserves EGT at near-fresh levels, while hot-air drying often yields small to moderate decreases that scale with temperature/time; in some studies, however, EGT remained largely unchanged after hot-air drying, underscoring the effects of interspecies and process parameters [39,40,42]. In *Lentinula edodes*, fresh mushrooms contained 1.02 ± 0.07 mg/g dry weight (d.w.) of EGT; processed samples decreased significantly to 0.58 ± 0.04 mg/g d.w. (*p* < 0.05), demonstrating EGT sensitivity to thermal treatment [41]. Overall, published data suggest that FD ≥ Fresh ≥ HD for EGT retention in most cases, with exceptions where slow air-drying can transiently elevate EGT.

The concentration of EGT in mushrooms shows substantial variability, primarily influenced by species- or strain-specific factors as well as by cultivation conditions, including substrate composition and environmental parameters [43]. Under identical growing conditions, two mushroom species may exhibit markedly different EGT concentrations, highlighting species identity as a critical variable in experimental design and nutritional profiling. Additionally, the substrate composition also significantly influences the EGT content. A study on *Pleurotus ostreatus* demonstrated that mushrooms cultivated on food–waste–based substrates accumulated nearly twice as much EGT as those grown on conventional sawdust-based media [44]. These findings are consistent with conclusions from the review by Tsiantas et al. (2021), which states that “differences in cultivation practices, including cultivation substrates, likely explain much of the large variation observed in the ergothioneine contents of both mushrooms and other foods” [45].

### 3.5. Enhancing Ergothioneine Biosynthesis

Several environmental and nutritional strategies have been demonstrated to effectively enhance EGT biosynthesis in cultivated mushroom species, providing promising approaches for enhancing their functional value in food and pharmaceutical applications. Supplementation with precursor amino acids—specifically methionine, cysteine, and histidine—has been shown to significantly increase EGT content in mushroom biomass. However, excessive concentrations (>0.5%) of these precursors may suppress mycelial growth and fruiting efficiency, indicating the need for dosage optimization [46]. The influence of species identity, cultivation parameters, and processing conditions on ergothioneine (EGT) content in mushrooms is shown in Table 3.

Light quality during mushroom development also plays a crucial role in the production of secondary metabolites. In particular, blue LED light (~450 nm) has been found to significantly increase EGT levels in *Pleurotus ostreatus* and *Lentinula edodes* when applied during fruiting body development, compared to both red LED and dark control conditions. In *L. edodes*, EGT content increased from 1.2 mg/g (control) to approximately 2.8 mg/g under blue light exposure [44,47]. Ergothioneine biosynthesis is also inducible under oxidative stress, as the molecule acts as a cytoprotective antioxidant that scavenges reactive oxygen species (ROS) and stabilises cellular redox balance. Experimental findings in *Agaricus bisporus* have demonstrated that cultivation conditions associated with increased oxidative load—such as high oxygen supply or the third flush of fruiting—are correlated with elevated EGT levels in fruiting bodies. This may reflect a stress-activated upregulation of the EGT biosynthetic gene cluster, functioning as a physiological response to mitigate environmental challenges [18].

## 4. Ergothioneine Extraction from Residuals and Purification

### 4.1. Extraction Principles

The physicochemical properties of EGT are fundamental to determining the most effective extraction strategies. EGT is characterized as a water-soluble, hydrophilic zwitterion compound [3,21], making aqueous and polar organic solvents particularly suitable for its extraction. A significant advantage for processing is EGT’s remarkable stability to heat and pH variations [2,21]. This inherent stability means that methods involving heat, such as hot-water extraction, can be employed without substantial degradation of the target compound, a critical consideration that differentiates EGT from many other heat-sensitive bioactive compounds [32,33]. Weng et al. (2024) [48] highlighted the advantages of ultrasound-assisted extraction (UAE) and microwave-assisted extraction (MAE) over conventional solvent methods, showing reductions in solvent use and extraction time while enhancing recovery of thermolabile compounds [48]. Hu et al. (2023) [49] demonstrated the efficiency of UAE for polyphenol recovery from *Boletus bicolor* under optimized conditions (42% ethanol, 34:1 mL/g solvent-to-material ratio, 41 min, 40 °C) [49]. While Soxhlet extraction remains a robust method, hybrid protocols are increasingly adopted to meet sustainability objectives. Sirohi et al. (2024) [50] reviewed the use of pulsed electric field (PEF) technology to recover bioactives from agro-industrial residues, where high-voltage pulses permeabilize cell membranes, releasing intracellular metabolites without compromising bioactivity [50].

### 4.2. Extraction Methods

Several methods are currently employed for EGT extraction from mushroom biomass, including the use of residuals. Hot water extraction is a widely adopted and effective method due to the water solubility and heat stability of EGT. For mushroom mycelium, hot water extraction—for example, subjecting mycelial suspensions to a 100 °C water bath for 5 min—has been shown to efficiently leach EGT into an aqueous solution [36]. For *Panus conchatus*, a similar approach involves resuspending cells in water and heat-treating them at 95 °C for 1 h [18]. Studies on *Pleurotus eryngii* products also indicate that hot water extracts yield higher EGT compared to ethanol extracts [25].

Solvent extraction, utilizing organic solvents such as ethanol or methanol, is also common, often in combination with water. For instance, 70% ethanol has been found to be more effective than pure solvents for extracting EGT and phenolic compounds from mushrooms like *Flammulina velutipes* [33]. Initial extraction from dried mushroom fruiting body powder can involve acetone or ethanol solutions [3,18,36]. Methanol is commonly used as an extraction solvent, particularly for highly sensitive analytical techniques such as UPLC-MS/MS [25,33].

High-Hydrostatic-Pressure Extraction (HHPE) is an advanced method that leverages high pressure to disrupt mushroom tissues, thereby facilitating the release and extraction of EGT. Optimised conditions for HHPE, such as 250 MPa pressure, 52 min extraction time, and distilled water as the solvent, have demonstrated high EGT yields (4.03 ± 0.01 mg/g d.w.) from *Pleurotus citrinopileatus* [32,33].

Submerged fermentation is a bioproduction method gaining traction for EGT synthesis. It involves cultivating specific mushroom species, such as *Panus conchatus*, in liquid media, leading to EGT accumulation primarily in the mycelia or fermentation broth [18,25], which offers significant advantages, including potentially higher yields, lower costs, and easier scalability compared to direct extraction from fruiting bodies or chemical synthesis [1,18,36]. The EGT is subsequently extracted from the mycelial biomass or fermentation liquid.

Ultrasound-assisted extraction (UAE) and microwave-assisted extraction (MAE) represent two major green extraction techniques that have been extensively applied to fungal systems [48,51]. However, when applied specifically to EGT extraction from *Pleurotus eryngii* and *P. citrinopileatus*, UAE showed limited efficiency compared to hot water or 70% ethanol extraction [52]. This indicates that, although the UAE offers advantages such as reduced solvent use and shorter extraction times, it may not be optimal for isolating water-soluble, low-molecular-weight thiols without further optimisation [51]. Similarly, MAE—despite its ability to shorten extraction times and lower solvent consumption—was found to yield less EGT than aqueous extraction in comparable systems [52]. These findings suggest that parameter tuning (e.g., irradiation power, exposure time, and temperature control) is critical to avoid thermal degradation of EGT.

Supercritical fluid extraction (SFE) using carbon dioxide has also been investigated as a green technology. While highly efficient for nonpolar compounds, SFE exhibits limited capacity to extract polar, water-soluble molecules, such as EGT. According to recent reviews, combining SFE with polar co-solvents, such as ethanol–water mixtures, could enhance recovery; however, quantitative comparisons with classical methods remain lacking [39]. Therefore, current evidence supports the use of SFE primarily as a complementary purification step rather than a standalone extraction strategy for EGT. The principal extraction techniques applied for ergothioneine recovery from mushroom biomass are shown in Table 4.

### 4.3. Purification Techniques

Following initial extraction, various purification techniques are employed to increase EGT purity. Ultrafiltration (UF), a membrane-based separation technique, is frequently used to purify EGT from aqueous solutions. Hollow fiber ultrafiltration membranes with specific pore sizes (e.g., 4 kDa or 6 kDa) can effectively separate EGT from larger molecules, leading to increased purity (e.g., 31.6% purity achieved with a 4 kDa membrane) [33,36].

Chromatography as a separation technique provides a range of methods indispensable for achieving high-purity EGT. Historically, techniques such as column chromatography, thin-layer chromatography, ion exchange chromatography, and paper electrophoresis have been utilised for purification from crude mushroom extracts [18,36]. More recently, specialised uniform particle ion exchange resins (e.g., Sunresin’s LX-1880) have been developed specifically for EGT fermentation liquids. These resins are designed for desalination, decolourization, and impurity removal, achieving removal rates of over 95% for inorganic and organic salts, as well as over 98% EGT recovery [25]. They are often integrated into advanced systems, such as simulated sequential moving Bed (SSMB) chromatography, for enhanced efficiency and recovery [25].

High-performance liquid chromatography (HPLC) is employed for both analysis and purification. EGT, being a hydrophilic zwitterion, is not optimally retained in traditional reversed-phase HPLC (RP-HPLC) without the use of ion-pairing reagents [3]. aqueous normal phase (ANP) HPLC, utilising columns like Cogent Diamond Hydride and a mobile phase of deionised water/acetonitrile/0.1% formic acid, offers a rapid, reliable, and sensitive method for EGT quantification and purification [3,35]. hydrophilic-interaction chromatography (HILIC) is also employed for separation in UPLC-MS/MS systems, typically using acetonitrile/formic acid as the mobile phase [25,33].

UPLC-MS/MS is a highly sensitive analytical technique used for precise identification and quantification of EGT, particularly in complex matrices such as cosmetics or biological samples [25,33]. UPLC-Q-TOF-MSE is also used to assess the purity of crude EGT extracts after combined HHPE and ultrafiltration/anion resin purification [33].

The primary objective of extraction and purification from residuals is to achieve high yields of EGT with high purity. For submerged fermentation, significant yields, such as 148.79 mg/L from *Panus conchatus* fermentation broth, have been reported, with established processes for obtaining crude EGT extracts [18]. Purity levels can be substantially enhanced through multi-step purification. Methods combining hot water extraction with ultrafiltration have demonstrated the capability to achieve EGT purity of up to 99.0% [36]. Optimised HHPE conditions have yielded an EGT content of 4.03 ± 0.01 mg/g dry weight [33]. A key challenge in EGT production, especially from fermentation broths, is the presence of high impurity and salt levels. Specialised ion exchange resins are being developed to overcome this, demonstrating high desalination and EGT recovery rates, which are crucial for cost-effective industrial production [25].

As mushroom processing generates various biomass streams (stems, spent mushroom substrate, culls) that are often considered waste, but are known to contain EGT and other valuable compounds like fibres, polysaccharides, and amino acids [33], this suggests a broader potential for valorisation than just EGT. Different extraction methods are highlighted for EGT, including hot water, ethanol, and HHPE [3,18,33,36]. For instance, 70% ethanol is effective for both EGT and phenolic compounds [33], while hot water is good for water-soluble substances including EGT [25].

In the context of sustainable processing, many researchers indicate that chromatographic purification is increasingly complemented by membrane-based separations and hybrid purification cascades. For example, combined ultrafiltration–ion-exchange systems have been optimised to minimise solvent use and reduce salt residues, achieving EGT recoveries exceeding 95% under mild operating conditions [39]. Furthermore, nanofiltration and electrodialysis are being explored as low-energy alternatives for desalination of fermentation broths, which remain a major cost factor in EGT downstream processing [53].

### 4.4. Optimisation of Extraction Conditions and Industrial Feasibility

Maximising recovery of ergothioneine and co-existing bioactives depends critically on optimising extraction parameters such as solvent type and polarity, temperature, duration, and sample pretreatments. Studies on chitosan extraction from mushroom stalks have revealed that freeze–thawing protocols improve yield and quality, resulting in up to a twofold increase in chitosan extraction and greater deacetylation degrees, which enhance antimicrobial properties [1]. Similarly, dual ethanol extraction protocols have been optimised for selective isolation of ergothioneine, lovastatin, and related compounds from fruiting bodies, balancing compound solubility and stability [24].

Hu et al. (2023) [49] applied response surface methodology (RSM) to ultrasound-assisted extraction of *Boletus bicolour*, determining that moderate ethanol concentrations (≈40%), short extraction times, and low temperatures maximise polyphenol yield while minimising energy input [49]. Prandi et al. (2023) [54] demonstrated that ultrasound-assisted methods significantly enhance the recovery of protein–polysaccharide complexes from fungal biomass compared to conventional aqueous extraction, due to cavitation-induced microstructural disruption [54]. Parí et al. (2025) [51] reviewed microwave-assisted protocols across multiple mushroom species, confirming that irradiation power, exposure time, and temperature critically affect both extraction efficiency and compound integrity [51].

**Figure 3:**
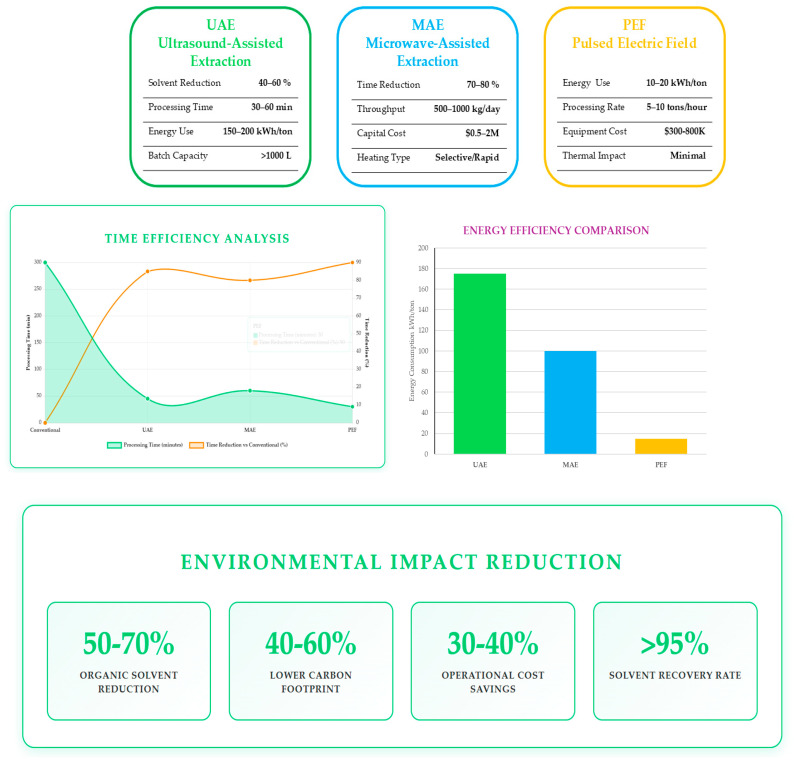
Comparative analysis of green extraction technologies for ergothioneine recovery.

The extraction and purification of ergothioneine from mushroom processing residuals have evolved from conventional solvent-based approaches to integrated, sustainable systems that combine thermal, non-thermal, and pressure-assisted methods. Research demonstrates the growing efficiency of hybrid and green extraction technologies—such as hot-water extraction, high-hydrostatic-pressure extraction, ultrasound, microwave-assisted processes, and pulsed electric field treatment—coupled with membrane separations and chromatographic purification, resulting in enhanced yield and purity. Optimisation through predictive modelling and the adoption of biorefinery concepts have enabled simultaneous valorisation of EGT and other valuable compounds (e.g., polysaccharides, β-glucans, chitosan, ergosterol, vitamin D_2_). The industrial feasibility of green extraction technologies is illustrated in Figure 3, which presents a comprehensive comparison of three advanced extraction technologies—Ultrasound-Assisted Extraction (UAE), Microwave-Assisted Extraction (MAE), and Pulsed Electric Field (PEF)—evaluated across key performance metrics.

The energy efficiency comparison (right) demonstrates PEF’s superior energy performance at 10–20 kWh/ton, representing a 92% reduction compared to conventional methods, while UAE and MAE consume 150–200 and 100 kWh/ton, respectively. The time efficiency analysis (left) illustrates processing time reductions of 80–90% across all three technologies compared to conventional extraction methods (300 min), with dual-axis visualization showing both absolute processing times and percentage improvements. The environmental impact reduction (bottom) quantifies the sustainability benefits, including a 50–70% reduction in organic solvent use, a 40–60% lower carbon footprint, 30–40% operational cost savings, and solvent recovery rates exceeding 95%, demonstrating the significant environmental advantages of these green extraction technologies for industrial-scale ergothioneine production.

### 4.5. Integration of Nano- and Molecular Encapsulation Technologies

To enhance the efficacy, stability, and bioavailability of ergothioneine and other bioactive compounds recovered from mushroom processing waste, advanced delivery systems, including nano- and molecular encapsulation, have been explored. Advanced delivery systems, including nano- and molecular encapsulation, enhance the efficacy, stability, and bioavailability of ergothioneine from mushroom waste. Layer-by-layer (LbL) electrostatic deposition produces edible coatings that combine fungal chitosan with vitamin D_2_, thereby controlling microbial spoilage in fresh-cut melons and fruit bars [1]. Nanoencapsulation stabilises bioactives and enables controlled release in functional foods. A straightforward chitosan matrix enrichment is sufficient for vitamin D, simplifying manufacturing [55]. Mushroom-derived bioactive peptides provide synergistic antioxidative, anti-inflammatory, and immune-modulating effects [56]. Muñoz-Tebar et al. (2023) [57] created antimicrobial edible films from fungal chitosan [57]. Weng et al. (2024) confirmed that matrices stabilise EGT in composite coatings [48]. Kataoka et al. (2025) [21] described circular food waste recycling during cultivation enhancing EGT circulation [21]. Wen et al. (2025) reported multifunctional systems co-delivering antioxidant, immunomodulatory, and anti-inflammatory agents [53]. Zhang et al. (2025) emphasised improved EGT bioavailability in complex matrices [31]. These technologies facilitate scalable, eco-efficient circular processes, maximising mushroom biomass utility while strengthening ergothioneine recovery within sustainable bioeconomy frameworks.

### 4.6. Challenges Related to Substrate Complexity

Mushroom residuals, particularly spent mushroom substrate (SMS), constitute complex mixtures that present specific technical challenges for ergothioneine extraction and purification. As a heterogeneous system containing lignocellulosic materials, fungal mycelium, residual nutrients, and diverse enzymes, SMS requires fractionation strategies to efficiently isolate target compounds [58]. The compositional heterogeneity introduces several processing complications. Different mushroom species produce SMS with varying compositions, creating systems that cannot be efficiently treated under identical extraction conditions [50]. This variability necessitates substrate-specific optimization of extraction parameters, including solvent polarity, temperature, and duration. The high fiber content, particularly neutral detergent fiber and acid detergent fiber in sawdust-based substrates, represents a principal barrier to efficient compound recovery [59,60].

The lignocellulosic matrix restricts mass transfer and solvent penetration, requiring either aggressive extraction conditions or enzyme-assisted approaches that hydrolyze cell wall constituents to facilitate bioactive release [51]. The complexity of components and their varied solubility profiles across different solvents has driven the development of multiple extraction strategies [53], yet conventional methods often necessitate excessive solvent use, elevated temperatures, and extended processing times that risk degrading thermolabile compounds [51].

Purification following extraction faces additional complexity challenges. The presence of co-extracted impurities can affect optimal processing conditions, such as pH-dependent enzyme activity [50]. Multi-stage purification—incorporating techniques such as ultrafiltration, ion-exchange chromatography, and dialysis—becomes essential to achieve pharmaceutical-grade purity, as discussed in Section 4.3. However, these enhanced purification steps can improve downstream processing; for instance, cellulose digestibility increases following bioactive extraction [59], supporting integrated biorefinery approaches. Addressing these complexity-related challenges requires process optimization tailored to specific SMS types and target compound characteristics, balancing extraction efficiency with bioactivity preservation and environmental sustainability.

## 5. Applications of Ergothioneine from Mushroom Residuals

### 5.1. Nutraceuticals and Functional Foods

Ergothioneine (EGT) is increasingly recognised as a valuable nutraceutical and a key component in functional foods, owing to its extensive health benefits beyond basic nutrition [1,18,23,35]. It is currently marketed as a dietary supplement, with products like ErgoFlex (OXIS International, Foster City, CA, USA) targeting specific health concerns such as chronic joint pain, cartilage health, and inflammation reduction [18,22]. EGT-rich mushroom extracts can be seamlessly incorporated into various supplements to impart potent antioxidant benefits. These applications aim to protect against oxidative stress, reduce the risk of cardiovascular diseases, and combat premature aging [8,20,22,25]. Beyond human consumption, EGT’s antioxidant properties make it a promising natural preservative in the food industry, particularly for aquatic products, where it can prevent lipid peroxidation and preserve colour, thereby extending shelf life [18,32].

When incorporated into vitamin D_2_-fortified edible coatings, EGT improves the oxidative stability of perishable foods and reduces microbial spoilage, prolonging shelf life [1]. Extracts rich in EGT and polysaccharides from mushroom byproducts exhibit prebiotic effects by stimulating the growth of beneficial lactic acid bacteria, thereby supporting gut microbiota balance and digestive health [21]. Fermentation of mushrooms with probiotic lactobacilli further increases EGT content and introduces anti-inflammatory properties, making them useful for functional and probiotic food formulations [61].

EGT surpasses conventional antioxidants through superior chemical stability across pH 3–10 and variable temperatures (unlike rapidly oxidizing vitamin C or UV-sensitive vitamin E), 100-fold cellular concentration via OCTN1-mediated transport, purely protective activity without pro-oxidant conversion in metal-rich environments, multi-mechanism action encompassing radical scavenging, metal chelation, mitochondrial DNA protection, and anti-inflammatory modulation, plus synergistic regeneration of vitamins C and E for enhanced formulation performance.

### 5.2. Cosmetics

EGT’s powerful antioxidant and anti-inflammatory properties have made it a highly sought-after ingredient in the rapidly expanding cosmetic industry [18,34]. It is widely utilized in formulations for anti-aging skincare and haircare products, as well as general beauty products and cosmeceuticals (e.g., creams, lotions, serums, masks) [18,34]. EGT contributes to improving skin firmness, vitality, and elasticity, and has demonstrated efficacy in mitigating signs of skin aging [18,34,35,62]. Its antimelanogenic properties also make it valuable for skin brightening applications [18].

Recent research further supports EGT’s multifunctional potential in cosmeceutical formulations. By modulating oxidative and inflammatory pathways, EGT protects dermal fibroblasts against photoaging and UV-induced damage, enhancing collagen synthesis and maintaining epidermal barrier integrity [39]. Edible chitosan-based films enriched with EGT or co-delivered antioxidants, such as vitamin D_2_, have demonstrated dual roles as protective packaging materials and bioactive skin-contact films, aligning with clean-label and circular economy trends [53,57].

### 5.3. Pharmaceutical and Therapeutic Uses

EGT is being explored as a significant potential therapeutic agent, with ongoing research into its pharmacological activities [18,20,35]. Its neuroprotective effects are particularly relevant for the prevention and potential treatment of neurodegenerative diseases such as Alzheimer’s. Studies show that combinations of EGT with other natural products like lactoferrin can exhibit synergistic neuroprotective effects [8,20,21,22,25,62].

EGT shows promise in addressing various oxidative stress-related diseases, including diabetes, ischemia–reperfusion injury, and liver diseases [34]. Its broader biological roles, including maintaining DNA biosynthesis, supporting normal cell growth, and enhancing cellular immunity, suggest a wider spectrum of pharmaceutical utility [18,36]. EGT can also mitigate nitrosative stress–induced lactoferrin inactivation, highlighting its role in preserving the activity of other beneficial compounds [20]. Increasing evidence from cellular and animal models confirms EGT’s potent antioxidant and cytoprotective effects. It neutralizes reactive oxygen species (ROS), limits lipid peroxidation, and maintains redox homeostasis, particularly in tissues prone to oxidative injury such as the liver, brain, and kidneys [16,19]. Its long tissue residence time and transporter-mediated uptake enhance its potential as a diet-derived therapeutic antioxidant [8]. Cellular and animal model studies indicate that ergothioneine concentrates in tissues susceptible to oxidative damage, such as the liver, brain, and kidneys, exerting cytoprotective effects. Its unique thiol/thione structure and specific transporter-mediated accumulation enable efficient defence against oxidative injury, supporting cellular redox homeostasis [19]. In vitro and in vivo evidence further suggests that ergothioneine’s ability to reduce lipid peroxidation and inflammation, key processes implicated in chronic diseases, is further supported. Its long tissue residence time and resistance to metabolic degradation enhance its therapeutic potential as a diet-derived antioxidant [16].

The selective uptake of ergothioneine by brain tissues via OCTN1 transporters, along with its immunomodulatory capabilities, supports its involvement in maintaining neuronal health [17]. Beyond antioxidation, ergothioneine exerts neuroprotective effects relevant to neurodegenerative disorders such as Alzheimer’s and Parkinson’s diseases. It modulates multiple pathological mechanisms, including oxidative stress, neuroinflammation, and mitochondrial dysfunction, which are implicated in neuronal loss. Its anti-inflammatory properties operate through the regulation of cytokines and the inhibition of proinflammatory pathways, contributing to the mitigation of systemic and neural inflammation. These mechanisms offer promise not only in neurodegeneration but also in a broader range of immune-related conditions. Ongoing research emphasizes the therapeutic value of integrating ergothioneine into dietary strategies aimed at disease prevention and health promotion [23].

### 5.4. Regulatory Status and Market Perspective

The commercial development and application of ergothioneine (EGT) depend not only on its biological efficacy but also on its regulatory acceptance across global markets. The regulatory assessments have formally recognised EGT as a safe and functional food ingredient, enabling its integration into a broad range of nutraceutical, cosmetic, and food formulations. The regulatory status of EGT in major jurisdictions, highlighting its authorisation as a Novel Food in the European Union, its GRAS (Generally Recognised As Safe) designation in the United States, and its evolving status under functional and import ingredient regulations in selected Asian markets is presented in Table 5.

The expanding regulatory acceptance, combined with EGT’s strong scientific profile and sustainable sourcing from mushroom residuals, positions it for substantial global market growth.

## 6. Economic Feasibility and Environmental Sustainability

### 6.1. Valorization of Waste and Circular Economy Principles

The mushroom industry, while economically significant, generates substantial volumes of organic waste, including spent mushroom substrate (SMS), stems, and culls. Traditionally, the disposal of these residuals incurs significant costs and poses a substantial environmental burden [18,21,31,32]. The strategic utilisation of these residuals as a source of high-value compounds, such as EGT, represents a direct application of circular economy principles. This approach transforms what was once a waste stream into valuable co-products, thereby reducing pollution, enabling resource reuse, and providing essential inputs for sustainable food systems [21].

Commercial cultivation of edible mushrooms generates a substantial volume of residual material, commonly referred to as spent mushroom substrate (SMS). After one or more fruiting cycles, the substrate—originally composed of lignocellulosic agricultural residues (e.g., straw, sawdust, manure) and fungal mycelium—becomes depleted of nutrients and is removed from production, often regarded as waste [58]. In the European Union, approximately 3.3 tonnes of SMS are generated per 1 tonne of mushrooms harvested, corresponding to ≈approximately 3.5 million tonnes of SMS annually [63]. On a global scale, this ratio translates to an estimated 242 million tons of SMS produced in 2022 [59,60]. These figures reveal that SMS is a significant material flow in fungal-agriculture systems, rather than a marginal by-product, underscoring its importance for valorisation and circular bioeconomy initiatives.

**Figure 4:**
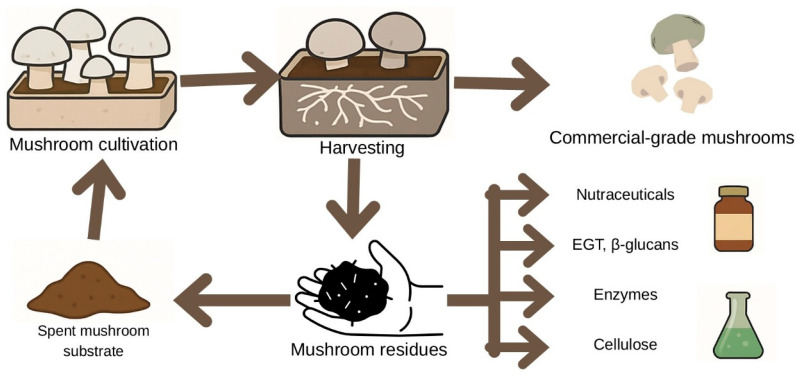
Schematic representation of the circular valorization pathway in mushroom production. The diagram illustrates the flow from cultivation and harvesting to the generation of commercial-grade mushrooms and residual biomass. Mushroom residues, including spent mushroom substrate, are redirected toward the recovery of bioactive compounds such as ergothioneine (EGT), β-glucans, enzymes, and cellulose, enabling their reintegration into nutraceutical, pharmaceutical, and agricultural applications within a circular bioeconomy framework.

Figure 4 presents a schematic representation of the circular valorisation pathway in mushroom production.

### 6.2. Cost-Effectiveness of EGT Recovery from Residuals

Historically, EGT production has been costly, with purification from natural sources reaching up to $1 million per kilogram. Although chemical synthesis has reduced the market price, it remains high [25]. This high cost underscores the need for more efficient and economical production methods. Fungus submerged fermentation, where EGT accumulates in mycelia, offers significant advantages for bioproduction, including high yield, lower cost, and easier scalability compared to direct extraction from fruiting bodies or complex chemical synthesis [1,18,25,36]. This method is particularly relevant as mycelia can be considered part of the “residual” biomass. The minimal infrastructure requirements for cultivating certain mushroom species, such as *Pleurotus* spp., which can effectively utilise various agricultural wastes as substrates, significantly reduce ex situ biomass-transport costs [21]. This localized, low-input cultivation model contributes substantially to the overall economic feasibility of EGT recovery. Optimising cultivation practices—such as using food waste as a primary substrate—has been shown to increase the EGT content in cultivated mushrooms [18,21], thereby directly enhancing the value of both the primary product and the residuals, and thus improving the overall return on investment.

## 7. Future Perspectives and Research Gaps

Despite significant advances in ergothioneine recovery from mushroom residuals, several critical challenges must be addressed for successful industrial implementation. The limitations are observed both in regulatory sector and technological feasibility.

A primary obstacle remains standardisation, as EGT content varies significantly across mushroom species, cultivation conditions, and processing methods. Establishing standardised protocols for substrate preparation, extraction parameters, and quality control metrics is essential for consistent industrial production. Current analytical methods, while precise, require harmonisation across laboratories to ensure reproducible quantification and purity assessment. Regulatory harmonisation across global markets represents another critical gap. While EGT has achieved GRAS status in the United States and Novel Food approval in Europe, varying regulatory frameworks complicate international commercialisation. Comprehensive toxicological studies, clinical trials demonstrating health benefits, and standardised safety assessments are needed to expand market access.

Also, scale-up challenges present both technical and economic barriers. It is common for laboratory-optimised extraction methods to exhibit reduced efficiency at industrial scales due to mass transfer limitations, heterogeneous feedstock composition, and equipment constraints. High-hydrostatic-pressure extraction and ultrasound-assisted methods, while promising, require substantial capital investment and energy optimisation for commercial viability. The transition from batch to continuous processing necessitates redesigning extraction workflows and developing robust process control systems.

For smooth technological integration within existing mushroom processing infrastructure, careful consideration of retrofitting costs versus greenfield investments is required. Developing modular extraction systems that can be easily integrated into existing production lines would accelerate their adoption. Additionally, life cycle assessments comparing different extraction technologies are lacking, making it difficult to quantify environmental benefits and guide sustainable technology selection.

Future research should prioritise: (1) developing real-time monitoring systems for EGT content during extraction, (2) establishing industry-wide quality standards and certification protocols, (3) optimising multi-product biorefinery approaches to improve economic feasibility, and (4) conducting pilot-scale demonstrations to validate techno-economic models and attract industrial investment.

## 8. Conclusions

The comprehensive analysis presented in this study highlights the potential of mushroom processing residuals as a sustainable and economically viable source of ergothioneine (EGT). EGT, a unique and highly stable thiol compound, plays a critical role in human health as a potent antioxidant with extensive neuroprotective, cardioprotective, and anti-aging properties. Its inability to be synthesised endogenously by humans, coupled with the existence of a dedicated cellular transporter, elevates its status to that of a “conditionally essential nutrient” or “longevity vitamin,” making dietary acquisition a must.

Mushrooms are the richest dietary source of EGT, and critically, their processing by-products—including stems, culls, and spent mushroom substrate—contain significant, often comparable, or even higher concentrations of this compound. The transition from viewing these by-products as waste to recognising them as high-value ingredients represents a significant shift towards a circular bioeconomy.

## Figures and Tables

**Table 1:** Ergothioneine content in various mushroom species and parts.

Mushroom Species	Mushroom Part/Type	Ergothioneine Content (mg/kg)	Source
*Pleurotus ostreatus* (*Oyster*)	fruiting body	11,800.00	[25]
*Pleurotus ostreatus* (*Oyster*)	fruiting body	118.91	[8,21]
*Pleurotus ostreatus* (*Oyster*)	fruiting body	2590.00	[33]
*Boletus edulis* (*King Bolete*)	fruiting body	528.14	[8,21]
*Boletus edulis* (*Porcini*)	fruiting body	7270.00	[18,34]
*Lentinula edodes* (*Shiitake*)	fruiting body	284.00	[8]
*Lentinula edodes* (*Shiitake*)	fruiting body	123	[25]
*Lentinula edodes* (*Shiitake*)	fermented product	1278.00–1775.00	[25]
*Flammulina velutipes* (*Enokitake*)	fruiting body	298.00	[8]
*Flammulina velutipes* (*Enokitake*)	fruiting body	151.00	[25]
*Cordyceps militaris*	fruiting body	382.00–799.00	[25]
*Cordyceps militaris*	mycelium	140.00	[25]
*Hericium erinaceus* (*Lion’s Mane*)	mycelium	376.20	[8]
*Agaricus bisporus* (*White Button*)	fruiting body	0.46	[8]
*Agaricus bisporus* (*White Button*)	fruiting body	210.00	[35]
*Agaricus bisporus* (*Brown*)	fruiting body	24.17	[13,35]
*Panus conchatus*	submerged fermentation broth (crude extract)	148.79 mg/L	[18]
*Pleurotus pulmonarius*	fruiting body (grown on sms)	2170.00	[18]
*Agaricus bitorquis*	mycelium (optimized conditions)	High	[36]
*Armillaria mellea* (*Honey Mushroom*)	mycelium	219.60	[37]
*Coprinus comatus* (*Shaggy Mane*)	mycelium	399.00	[38]
*Coriolus versicolor* (*Turkey Tail*)	mycelium	13.00	[38]
*Ganoderma lucidum* (*Reishi*)	mycelium	16.50	[38]
*Pleurotus eryngii* (*King Oyster*)	fruiting body	Fruiting Body	[25]
*Pleurotus eryngii* (*King Oyster*)	mycelium (regular)	Mycelium (regular)	[25]
*Pleurotus eryngii* (*King Oyster*)	mycelium (hi-ergo)	Mycelium (Hi-Ergo)	[25]
*Pleurotus ostreatus* (*Oyster*)	culls	Culls	[21,33]
*Lentinula edodes* (*Shiitake*)	culls	Culls	[32,33]
*Agaricus bisporus* (*White Button*)	stem waste	Stem Waste	[33]

**Table 2:** Effects of drying method on ergothioneine content in mushrooms.

Mushroom Species	Drying Method	Result on EGT	Source
*Pleurotus ostreatus* (oyster mushroom)	HAD (40 °C) vs. FD	Highest EGT retention among tested methods.	[40]
*Pleurotus citrinopileatus* (golden oyster mushroom)	ND (natural ventilation), FD, HD	EGT increased to 4.03 ± 0.01 mg/g d.w., higher than FD and HD.	[33]
*Cyttaria espinosae* (wild “digüeñe” mushroom)	FD, HAD	EGT not detected; negligible retention under HAD.	[40]
Various species (*Auricularia* spp., *Pleurotus* spp.)	Review of HAD, solar and microwave drying	EGT content is largely unchanged under moderate HAD.	[39]
*Lentinula edodes* (shiitake)	HAD + pasteurization	EGT decreased from 1.02 ± 0.07 to 0.58 ± 0.04 mg/g d.w., indicating heat sensitivity.	[41]
*Pleurotus ostreatus* (oyster mushroom)	HAD (40 °C) vs. FD	Highest EGT retention among tested methods.	[40]

**Table 3:** Environmental and nutritional factors modulating ergothioneine biosynthesis in cultivated mushrooms.

Factor	Experimental Description/Observation	Effect on EGT Content	Representative Species/Model	Source
Species and strain	Comparative analysis of multiple mushroom species under identical growth conditions.	Significant interspecific variation in EGT concentration, reflecting species-specific biosynthetic capacity.	*Agaricus bisporus*, *Pleurotus ostreatus*, *Lentinula edodes*	[43]
Substrate composition	Use of food-waste–based substrate versus conventional sawdust medium.	EGT content nearly doubled in mushrooms cultivated on food-waste substrate.	*Pleurotus ostreatus*	[44]
Amino acid supplementation	Addition of histidine, methionine, cysteine, yeast extract, and peptone to growth media.	Marked enhancement of EGT biosynthesis; excessive (>0.5%) precursor concentration reduced growth rate.	*Agaricus* spp.	[47]
Light quality	Exposure to blue LED light (~450 nm) during fruiting body formation.	Increased EGT from 1.2 mg/g to ~2.8 mg/g dry weight; elevated antioxidant activity.	*Lentinula edodes*, *Pleurotus ostreatus*	[44,47]
Oxidative stress induction	Cultivation under elevated oxygen or late fruiting flush (3rd harvest).	Elevated EGT levels associated with oxidative stress response.	*Agaricus bisporus*, *Panus conchatus*	[18]
Drying and processing conditions	Comparison of HAD, FD, ND, and pasteurization treatments.	EGT retention pattern: FD ≥ Fresh ≥ HAD; HAD + pasteurization caused significant losses.	*Pleurotus* spp., *Lentinula edodes*	[39,41]

**Table 4:** Ergothioneine extraction and purification methods.

Method Type	Principle	Mushroom Source	Reported Yield/Purity	Advantages/Disadvantages	Source
Hot Water Extraction	Mycelial suspension in 1:40 water, 100 °C for 5 min (no stirring).	*Pleurotus ostreatus* mycelium	1700 mL EGT aqueous solution (from 50 g mycelium)	Simple, low-cost, exploits thermal stability of EGT; limited selectivity.	[48,50]
Hot Water Extraction	Cells resuspended in water, 95 °C for 1 h.	*Panus conchatus* fermentation broth	Crude extract obtained	Efficient for crude extracts; maintains antioxidant integrity; may co-extract impurities.	[48,50]
Solvent Extraction (Ethanol)	Pulverized mushroom sonicated 30 min in ethanol and filtered.	*Agaricus bisporus*, *Lentinula edodes*, *Pleurotus ostreatus*	Better analytical signal than water extraction.	Effective for EGT + phenolics; easy lab-scale recovery; solvent removal required.	[49,50]
Solvent Extraction (70% Ethanol)	Extraction using 70% ethanol (*v*/*v*).	*Flammulina velutipes*	2.05 ± 0.18 mg g^−1^ DW (EGT).	Maximizes recovery of polar + semi-polar metabolites; green-solvent alternative.	[48,50]
High-Hydrostatic-Pressure Extraction (HHPE)	250 MPa, 52 min, distilled water (1:10 liquid–solid ratio).	*Pleurotus citrinopileatus*	4.03 ± 0.01 mg g^−1^ DW (highest EGT content).	Disrupts tissues effectively, enhances mass transfer; high energy demand.	[48,50]
Ultrasound-Assisted Extraction (UAE)	Sonication of powdered biomass (20–40 kHz) to enhance solvent penetration.	*Boletus bicolor* (model species)	Polyphenols ↑; EGT retention confirmed.	Accelerated diffusion, mild temperature, scalable green method.	[49,50]
Submerged Fermentation	Liquid culture with molasses, soy peptone, and amino-acid precursors (His, Met, Cys).	*Panus conchatus*	148.79 mg L^−1^ (highest EGT concentration).	High yield, low cost, easily scalable; downstream purification required.	[49,50]
Ultrafiltration (UF)	Hollow-fiber membrane (4 kDa or 6 kDa cut-off) for crude filtrate fractionation.	Mycelial fermentation liquid	31.6% purity (4 kDa membrane).	Simple, low-energy separation; limited resolution for small molecules.	[48]
Ion-Exchange Chromatography (Specialized Resin)	LX-1880 uniform-particle resin under SSMB system.	EGT-rich fermentation liquids	>95% desalination, >98% EGT recovery.	High selectivity for charged species; industrial scalability; resin cost.	[48]
Traditional Chromatography	Column chromatography, TLC, ion-exchange, and paper electrophoresis.	*Colibus genus*, *Agrocybe aegerita* (tea tree mushroom)	High purity fractions obtained.	Robust, well-established; labor-intensive, multi-step.	[48]
HPLC (ANP Mode)	Cogent Diamond Hydride column, DI water/acetonitrile + 0.1% formic acid; UV 254 nm.	*A. bisporus*, *L. edodes*, *P. ostreatus*	96.5–100.3% recovery (R^2^ = 0.99999).	Rapid, reproducible quantification of EGT in complex matrices.	[48]
UPLC–MS/MS (HILIC)	Methanolic extract analysed by HILIC–triple quadrupole MS.	Cosmetics samples (applicable to mushroom extracts)	LOD 25–50 μg kg^−1^; R^2^ > 0.999.	High sensitivity and specificity for EGT; cost-intensive instrumentation.	[48]

**Table 5:** Regulatory status of ergothioneine in major markets.

Region	Regulatory Category	Status for EGT	Key Document
European Union	Novel Food ingredient	Synthetic L-ergothioneine is authorised as a Novel Food ingredient under Regulation (EC) No 258/97 and later under Regulation (EU) 2015/2283.	Commission Implementing Decision (EU) 2017/1281—Authorising placing on the market of synthetic L-ergothioneine as a Novel Food ingredient. EUR-Lex
United States	GRAS (Generally Recognised As Safe)	EGT notified to the U.S. FDA as GRAS for specified food uses.	FDA GRAS Notice Response Letter (GRN 1191) for ergothioneine. U.S. Food and Drug Administration
Asia (examples)	Novel Food/Import Ingredient Control	In Asian markets (e.g., China), EGT is regulated as an import-controlled functional ingredient; specific national standards vary.	“Ergothioneine’s Regulatory Status in China.” Zmuni Industry News

## Data Availability

No new data were created or analyzed in this study. Data sharing is not applicable to this article.
